# A new species of sisorid catfish of the genus *Exostoma* from the Salween drainage, Yunnan, China

**DOI:** 10.24272/j.issn.2095-8137.2017.065

**Published:** 2017-09-18

**Authors:** Xiao-Yong Chen, William J. Poly, Catania David, Wan-Sheng Jiang

**Affiliations:** ^1^Southeast Asia Biodiversity Research Institute, Chinese Academy of Sciences, Yezin Nay Pyi Taw 05282, Myanmar; ^2^Kunming Institute of Zoology, Chinese Academy of Sciences, Kunming Yunnan 650223, China; ^3^Department of Ichthyology, California Academy of Sciences, 55 Music Concourse Drive, San Francisco CA 94118, USA

**Keywords:** Glyptosterninae, Sisoridae, Nujiang, Gaoligong Mountain, Yunnan

## Abstract

A new species of the sisorid catfish genus *Exostoma* Blyth, 1860 was collected from two hill-stream tributaries of the Nujiang (Salween River) drainage in Gaoligong Mountain, south-western Yunnan Province, China from 2003 to 2006 and from two tributaries of the Salween River in Cangyuan County, Lingcang Prefecture, Yunnan Province, China (in 2007) and in Yongde County, Lingcang Prefecture, Yunnan Province, China (in 2015). *Exostoma gaoligongense* **sp. nov.** is the 10th species of the genus and is most similar to *E*. *vinciguerrae* in morphology but can be distinguished by pelvic fin reaching anus vs. not reaching; maxillary barbels just reaching or slightly surpassing pectoral-fin origin vs. surpassing pectoral-fin origin or even reaching posterior end of gill membrane; abdominal vertebrae 23-25 vs. 25-27; length of dorsal fin/dorsal to adipose distance 90.3%-287.0% vs. 59.2-85.7. A key to *Exostoma* spp. is provided.

## INTRODUCTION

The Sisoridae is the largest family of Asian catfish, with more than 200 species and 22 genera (Ferraris, 2007; Ng, 2015). Members are found along the entire southern arc of the Asian continent and comprise a significant portion of the hill-stream fauna in southern and eastern Asia (Ng & Jiang, 2015). Recent morphological (Ng, 2015) and molecular research (Ng & Jiang, 2015) reconstructed the monophyly of Sisoridae and divided it into Sisorinae and Glyptosterninae subfamilies. The Glyptosterninae is well-supported as a monophyletic group with 15 synapomorphies, within which *Exostoma* is monophyletic and considered to be a sister group of *Pseudexostoma* and *Oreoglanis*, with 11 synapomorphies (Ng, 2015), or a sister group of *Glyptosternon* (Ng & Jiang, 2015).

*Exostoma* Blyth, 1860 is a genus of the subfamily Glyptosterninae with nine species occurring in the Brahmaputra, Chao Phraya, Irrawaddy and Salween River drainages in China, India, Myanmar (=Burma) and Thailand (Chu et al., 1990; Hora & Silas, 1952; Lalramliana et al., 2015; Ng & Vidthayanon, 2014; Tamang et al., 2015; Vishwanath & Joyshree, 2007; Wu & Wu, 1992). The genus is diagnosed by: continuous post-labial groove; gill openings not extending onto venter; homodont dentition; oar-shaped, distally flattened teeth in both jaws; tooth patches separated in upper jaw; 10–11 branched pectoral rays (Thomson & Page, 2006).

The broadest taxonomic treatment of the genus *Exostoma* along with related genera was that of Hora & Silas (1952). At that time there were 14 nominal species of *Exostoma*, most of which are included in other genera now, and of the 14, five nominal species belong in the genus *Exostoma*: *E*. *berdmorei* Blyth, 1860 (type-species), *E*. *labiatum* (McClelland, 1842), *E*. *stuarti* (Hora, 1923), *E*. *chaudhurii* (Hora, 1923), and *E*. *vinciguerrae* Regan, 1905. *Exostoma chaudhurii* was considered a junior synonym of *E*. *vinciguerrae* by Norman (1925) and followed by Thomson & Page (2006) and others. Later, five new species were described: *E*. *barakensis* Vishwanath & Joyshree 2007; *E*. *effrenum* Ng & Vidthayanon 2014; *E.* *peregrinator* Ng & Vidthayanon 2014; *E*. *sawmteai* Lalramliana, Lalronunga, Lalnuntluanga & Ng 2015; *E*. *tenuicaudata* Tamang, Sinha & Gurumayum 2015. The name *Exostoma barakensis* was formed incorrectly and should be spelled *E*. *barakense* (an adjective based on the Barak River; see Etymology of the new species below). There are two nominal species recorded in China: *E*. *labiatum* from upper Brahmaputra and Irrawaddy drainages (Chu & Mo, 1999), and *E*. *vinciguerrae* from Irrawaddy drainage (Zhu, 1995). The records in China are considered as the same species under the name of either *E*. *labiatum* (Chu et al., 1990; Chu & Mo, 1999; Chen, 2013) or *E*. *vinciguerrae* (Zhu, 1995).

On 7 October 2003, the authors collected some sisorid catfish, representing an undescribed species, from a hill-stream tributary of the Nujiang (=upper Salween River) in Gaoligongshan National Nature Reserve. Additional specimens were captured in the same stream on 25 April 2004 and 6 May 2006, and an adjacent stream on 8 May 2006. Specimens obtained from Xinya River, a tributary of Nanting River on 16 February 2007 and Nanzha River, a tributary of Nangun River on 9 February 2015 were considered as the same species. We provide a description of the new species and compare it to other *Exostoma* spp.

## MATERIALS AND METHODS

Fishes were collected with an electroshocker or purchased from local fishermen, fixed in 10% formalin, and transferred to 75% ethanol for long-term storage, or fixed in 75%–100% ethanol. Measurements were made point to point with digital calipers and recorded to 0.1 mm. Counts and measurements of paired structures were made on the left side of specimens, except counts of pectoral-fin rays were on both sides when available. Counts and measurements followed Hubbs & Lagler (1958) and Ng & Kottelat (1999). Vertebral counts were made from radiographs following the methods of Roberts & Ferraris (1998). Morphology of teeth on jaws follows Steinitz (1961). Fishes were cataloged in the Kunming Natural History Museum of Zoology, the Kunming Institute of Zoology, the Chinese Academy of Sciences, Kunming, Yunnan, China (KIZ) and in the California Academy of Sciences, San Francisco, California, USA (CAS). Symbolic codes for institutions are those given by Leviton et al. (1985). Some comparative specimens were kept in the Southeast Asia Biodiversity Research Institute (SEABRI), Chinese Academy of Sciences, Nay Pyi Taw, Myanmar.

## RESULTS

### *Exostoma gaoligongense* sp. nov.

Figures 1, 2

**Figure 1:**
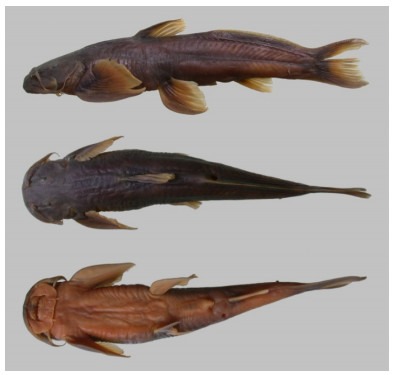
Holotype of *Exostoma gaoligongense* sp. nov. (KIZ 200310738); lateral (top), dorsal (middle), and ventral (bottom) views (photos by Xiao-Yong Chen)

**Figure 2:**
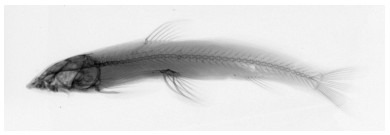
Radiograph of *Exostoma gaoligongense* sp. nov. (paratype CAS 220955) (rotated horizontally)

English common name: Gaoligong mountain catfish

Chinese common name: Gao Li Gong Yan (高黎贡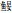)

**Holotype**: KIZ 200310738 (0699), 65.5 mm SL, Manggang River (芒岗河, N25°16'38.3", E98°48'03.6", 1 270 m a.s.l.), a tributary of Nujiang (=Salween River), Baihualing Village, Mangkuang Township, Baoshan City, Yunnan Province, China, 7 October 2003, collected by David Catania, William J. Poly, Xiao-Yong Chen, Jing-Hui Chen, et al.

**Paratypes**: KIZ 200310737 (0698), 200310740 (0700), CAS 220955 (ex KIZ 200310739), 3 ex., 57.0–74.5 mm SL, collected with the holotype. KIZ 20040425001 (05239), 20040425002 (05240), 20040425003 (05241), 20040425004 (05242), 20040425008 (05244), CAS uncat. (05237, 05238, 05243), 8 ex., collected at the type locality, 25 April 2004, collected by Xiao-Yong Chen, Jian Yang, Fei Wu, Jing-Hui Chen. KIZ DAN06–107 (07371–07376), 6 ex., 64.1–76.3 mm SL, collected at Manggang River, 6 May 2006, collected by David Neely, Xiao-Yong Chen, Xiao-Fu Pan, Yan-Fei Huang, Rui Min. KIZ DAN06–110 (M1–M6, 07384), 7 ex., 55.8–77.3 mm SL, collected at Tangxi River (烫习河, N25°18'25.7", E98°48'37.3", 1 143 m a.s.l.), a tributary of Nujiang, Baihualing Village, Mangkuang Township, Baoshan City, Yunnan Province, China, 8 May 2006, collected by David Neely, Xiao-Yong Chen, Xiao-Fu Pan, Yan-Fei Huang, Rui Min.

**Non-types**: 3 juveniles (KIZ uncat.), 33.8–39.0 mm SL, collected with the holotype on 7 October 2003; KIZ DAN06–107 (07377, 7), 50.7–76.9 mm SL, collected at Manggang River on 6 May 2006; KIZ DAN06–110 (77 uncat.), 36.6–66.6 mm SL, collected at Tangxi River on 8 May 2006; KIZ 20070012, 14, 15, *n*=21, 3 ex., 50.0–61.0 mm SL, collected at Xinya River (新牙河, N23°16'26.10", E99°07'34.01", 823 m a.s.l.), a tributary of Salween, Cangyuan County, Lingcang Prefecture, Yunnan Province, China, 16 February 2007, collected by De-Ping Kong. KIZ 2015001065, 66, 68–70, 74, *n*=10, 6 ex., 48.1–54.1 mm SL, collected at Nanzha River (南榨河, N23°45'37.44", E99°19'0.84", 790 m a.s.l.), a tributary of Salween, Junsai Township, Yongde County, Lingcang Prefecture, Yunnan Province, China, 9 February 2015, collected by Jun-Xing Yang.

**Diagnosis**: Adipose fin confluent with caudal fin, caudal fin emarginate, pectoral fin extending to vicinity of dorsal-fin origin, pelvic fin reaching anus, gill opening extending to ventral surface of the body, nasal barbels reaching or surpassing anterior edge of eye, maxillary barbels just reaching or slightly surpassing pectoral-fin origin, branched pectoral-fin rays 9–11, abdominal vertebrae 23–25, snout length 39.7%–53.3% HL, length of dorsal-fin base 8.3%–12.1% SL, length of anal fin 13.1%–15.8% SL, length of adipose-fin base 31.7%–45.2% SL, caudal-peduncle length 18.8%–22.3% SL, caudal-peduncle depth 7.3%–10.8% SL, body depth at anus 11.3%–16.9% SL, interorbital width 27.5%–35.1% HL, length of dorsal fin/dorsal to adipose distance 90.3%–287.0%, anal to caudal distance/pelvic to anal distance 88.2%–116.7%.

**Description**: Morphometric and meristic data are given in Table 1. Head and body slightly rounded and depressed. Head medium size, with minute papillae scattered on dorsal and lateral surfaces and on maxillaries. Snout blunt and depressed. Rostral cap with non-prominent groove in middle of anterior edge, turning to pelvic side and forming prominent preoral groove with papillae on surface. Mouth inferior, opening transversely. Tooth bands on upper and lower jaws visible when mouth in normal position or when made to close. Premaxillary with two semicircular-shaped tooth bands, contacting at posteromedial margins. Dentary with two distinctly separate tooth bands. Oar-shaped teeth prominent on both jaws, and pointed conical small teeth along the posterior borders of the teeth patches. Lower lip thin, flat, split into two big lateral lobes and two small median lobes by notches, posterior margin without fimbriate projections. Postlabial groove on lower jaw present, not interrupted in middle. Middle lobe of upper lip with papillae. Pelvic side of rostral barbel and lateral lobe of lower lip with feather-like wrinkles. Anterolateral portion of lateral lobe of lower lip connected with maxillary barbel through a membrane. Eyes tiny, subcutaneous, on dorsal side of head. Distance from posterior edge of eye to upper corner of gill opening almost equal to distance from posterior edge of eye to posterior edge of anterior nostril. Four pairs of flattened barbels. Nasal barbels long, in mid-point from anterior edge of eye to snout tip, tips reaching middle of or surpassing posterior edge of eye. Maxillary barbels broadly connected to lower part of head by fold of skin, tips just reaching or slightly surpassing pectoral-fin base. Mandibular barbels, two pairs. Outer mandibular barbels with rough surface, situated on underside of head, bases covered by lower lip, projecting laterally, longer than inner pair. Inner mandibular barbels short, in notches along posterior margin of lower lip. Upper corner of gill opening level to ventral side of eye, lower corner extending to ventral side of pectoral fin.

**Table 1:** Meristic and morphometric characters of *Exostoma gaoligongense* sp. nov. (*n*=25)

**Characters**	**Holotype**	**Range**	**Mean**	***SD***
Dorsal-fin rays	ⅰ, 5	ⅰ, 5		
Pectoral-fin rays	ⅰ, 11	ⅰ, 9 (*n*= 6), 10 (*n*= 11), 11 (*n*= 8)		
Pelvic-fin rays	ⅰ, 5	ⅰ, 5		
Anal-fin rays	ⅰ, 4	ⅰ, 4		
Principal caudal-fin rays (upper+lower=total)	8+9=17	8+9=17 (*n*=24), 8+8=16 (*n*=1)		
Vertebrae		23–25+15–17=39–42 (*n*= 25)		
Total length	79.4	50.2–91.6	76.3	10.7
Standard length (SL)	65.5	41.4–77.3	63.5	9.1
Head length (HL)	13.4	9.3–15.0	13.3	1.4
**Percentage of SL (%)**				
Body depth	18.3	13.0–19.2	16.6	1.6
Body depth at anus	16.2	11.3–16.9	14.2	1.6
HL	20.5	18.7–23.9	21.0	1.3
Head depth	13.6	9.4–13.9	12.4	1.1
Predorsal length	40.8	28.6–43.3	39.1	2.9
Preventral length	44.9	36.6–49.1	45.3	2.5
Preanal length	72.2	64.7–75.1	72.4	2.0
Caudal-peduncle length	21.5	18.8–22.3	20.8	0.8
Caudal-peduncle depth	10.8	7.3–10.8	9.2	1.0
Length of dorsal-fin base	11.3	8.3–12.1	10.1	1.0
Length of adipose-fin base	36.5	31.7–45.2	37.0	3.6
Length of anal-fin base	6.6	4.1–7.3	6.0	0.7
Dorsal to adipose distance	15.7	6.3–18.6	14.0	3.3
Length of dorsal fin	18.3	15.3–19.8	17.8	1.3
Length of anal fin	14.0	13.1–15.8	14.5	0.9
Length of pectoral fin	24.1	21.9–25.9	24.1	1.2
Length of pelvic fin	18.9	16.9–20.4	19.0	0.9
Length of caudal fin	21.1	18.3–23.0	20.6	1.3
Pectoral to pelvic distance	28.1	26.9–31.6	29.7	1.2
Pelvic to anal distance	27.3	23.3–29.4	27.1	1.8
**Percentage of HL (%)**				
Snout length	46.3	39.7–53.3	47.3	3.9
Head width	102.2	91.3–112.4	100.5	5.2
Head depth	66.4	49.8–66.4	58.8	4.2
Eye diameter	11.9	11.0–16.9	13.6	1.6
Mouth width	44.8	37.9–53.9	46.2	5.1
Interorbital width	35.1	27.5–35.1	30.8	2.0
Caudal-peduncle length/depth	2.0	1.9–2.8	2.3	0.3
Length of dorsal fin/dorsal to adipose distance (%)	116.5	90.3–287.0	136.8	46.6
Length of pectoral fin/pectoral to pelvic distance (%)	85.7	73.4–96.3	81.3	4.7
Length of pelvic fin/pelvic to anal distance (%)	69.3	58.6–83.1	70.6	6.2
Length of anal fin/anal to caudal distance (%)	50.0	49.6–62.5	54.3	3.5
Pectoral to pelvic distance/pelvic to anal distance (%)	103.0	92.7–134.7	110.3	10.9
Anal to caudal distance/pelvic to anal distance (%)	102.8	88.2–116.7	99.3	8.3

Predorsal part of body rounded and deep, back slightly convex, abdomen flat, smooth, without papillae, gradually compressed posteriorly. Pectoral fin round, first ray broad, flattened with numerous plicae on ventral surface, tip of fin nearly reaching to, reaching to, or extending beyond dorsal-fin origin. Dorsal fin without spine, distal margin truncate, its origin above tip of pectoral fin. Predorsal length greater than distance from dorsal-fin origin to adipose-fin origin. Tip of depressed dorsal fin not reaching adipose-fin origin. Adipose fin long, its origin above or slightly anterior of anus, confluent with caudal fin. Pelvic-fin origin under fourth branched dorsal ray, its tip sometimes reaching anus, first ray broad, flattened with numerous plicae on ventral surface. Anus and urogenital opening about two-thirds distance from pelvic-fin insertion to anal-fin origin. Anal fin distal margin rounded slightly. Distance from anal-fin origin to caudal-fin base almost equal to distance from anal-fin origin to pelvic-fin origin. Caudal fin emarginate, lower lobe slightly longer than upper lobe. Lateral-line complete, originating at upper corner of gill opening, arching slightly upward above pectoral-fin base, sloping downward until pelvic-fin origin (on holotype), then curving upward slightly pelvic-fin base (on holotype), thereafter straight on midline of body and of caudal peduncle.

**Coloration of live and preserved specimens**: Head and back gray. Gill membrane transparent. Body gray above lateral-line, lighter below lateral-line lighter on caudal peduncle, abdomen yellowish. Non-prominent black stripe along dorsal mid-line and lateral-line. Dorsal-fin membrane yellow and rays light gray. Adipose fin dark gray at base, transparent and yellowish in the margin. Pectoral-fin and pelvic-fin bases darker, fin rays yellow and transparent at the edge. Unbranched dorsal-fin and anal-fin rays grayish. Caudal-peduncle base black, posterior margin of the black blotch concave, middle part of caudal fin yellow, distal part dark gray. Margin of upper and lower lobes gray. The grayish coloration of live specimens changes to brownish tones after preservation.

**Allometry**: Juveniles smaller than 39.0 mm SL have a more depressed head; smaller eye, eye diameter 6.7%–8.2% HL vs. 11.0%–14.3%, interorbital width 4.5–4.8 times of eye diameter vs. 2.1–2.9; bigger head, head width much larger than body width vs. head width almost equal to body width; longer adipose-fin base and origin of adipose fin more anterior, consequently distance between dorsal fin and adipose fin is shorter; longer pectoral fin, its tip surpasses dorsal-fin origin; slender caudal peduncle, caudal-peduncle length 2.6–2.8 times its depth vs. 1.9–2.2.

**Figure 3:**
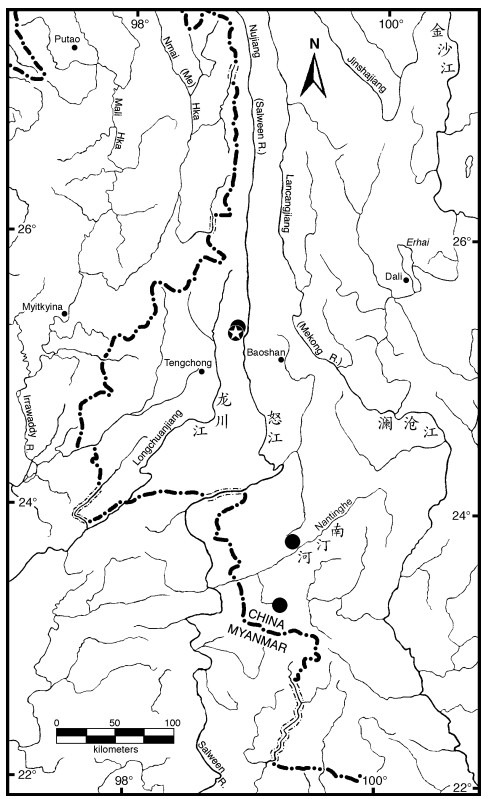
The region of western Yunnan Province, China and northeastern Myanmar showing the four collections sites

**Figure 4:**
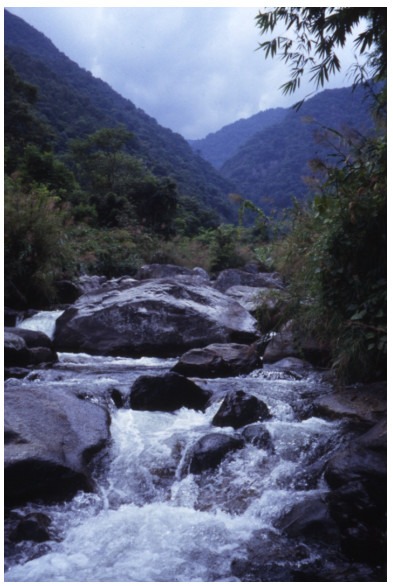
Manggang River at the type locality of *Exostoma gaoligongense* sp. nov. (photo by David Catania)

**Distribution**: Currently only known from four tributaries of the Salween River drainage in Yunnan, China (Figure 3), including the Manggang River (Figure 4) and Tangxi River, tributaries of Nu River (Nujiang), Xinya River, a tributary of Nanting River, and Nanzha River, a tributary of Nangun River. Probably also exists in other mountain streams in east slope of Gaoligong Mountain that drain into the Nujiang. The four known locations are close to China-Myanmar border, it hopefully also occurs in Salween Drainage of Myanmar.

**Etymology**: The specific name is an adjective that refers to the Gaoligong Mountain in which the type locality is located, and the suffix agrees in gender with the generic name *Exostoma* (gender neuter).

**Notes on biology**: This species was collected from shallow water ( < 1 m deep) in a fast flowing stream with clear water. Water temperature was 18.8 ℃, water pH 6.95, conductivity 45.6 μS/cm. The bottom substrate was boulders, cobbles, gravel, and sand with many diatoms that made the rocks slippery. This species was obtained from fast water and small waterfalls. The new species of *Exostoma* seems to have much lower tolerance to either low dissolved oxygen or to stress from electrofishing than *Pseudexostoma brachysoma* Chu, 1979, which occurs in the same habitat. After shocking sampling on 7 October 2003, all the *Exostoma* were dead, whereas all the individuals of *P*. *brachysoma* survived until the next morning.

**Associated species**: Only four species were collected at the Manggang River: *Gymnodiptychus integrigymnatus* Huang 1981, *Schistura* *poculi* (Smith) 1945, *P*. *brachysoma*, and *Exostoma gaoligongense*. On 7 October 2003 at a downstream site in the Manggang River, we also collected four species: *Schizothorax lissolabiatus* Tsao, 1964, *Misgurnus auguillicaudatus* (Cantor) 1842, *Schistura longa* (Zhu) 1982, and *Channa gachua* (Hamilton) 1822.

## DISCUSSION

*Exostoma gaoligongense* is similar to *E*. *berdmorei*, *E*. *peregrinator*, *E*. *sawmteai* and *E*. *vinciguerrae* by sharing adipose fin confluent with caudal fin vs. free from it in *E*. *barakense*, *E*. *effrenum*, *E*. *labiatum*, *E*. *stuarti* and *E*. *tenuicaudata*. *Exostoma gaoligongense* and *E*. *vinciguerrae* can be distinguished from *E*. *berdmorei*, *E*. *peregrinator* and *E*. *sawmteai* by gill opening extending to ventral surface of the body vs. not reaching, and caudal fin emarginate vs. lunate or forked. Pelvic fin reaches anus in *E*. *gaoligongense*, *E*. *labiatum*, *E*. *peregrinator*, *E*. *berdmorei*, *E*. *effrenum* vs. not in *E.* *vinciguerrae*, *E*. *sawmteai*, *E*. *barakense* and *E*. *tenuicaudata*.

**Figure 5:**
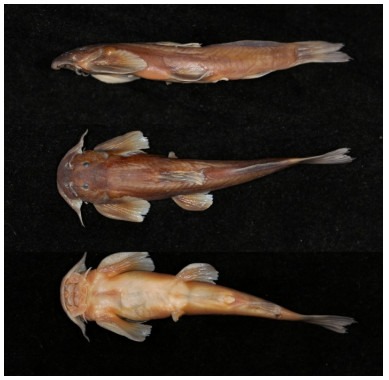
*Exostoma vinciguerrae* (SEABRI–20150257), 56.9 mm SL, from Zeyar Stream, Hponkanrazi Wildlife Sanctuary, 9 Dec., 2015; lateral (top), dorsal (middle), and ventral (bottom) views (photos by Xiao-Yong Chen)

*Exostoma gaoligongense* is most similar to *E*. *vinciguerrae* (Figure 5) for similar body shape, color pattern, and overlapped fin ray counts, number of vertebrae, and metric characters, but the former can be distinguished from the latter by the following characters: pelvic fin reaching anus vs. not reaching; pectoral fin slightly longer, extending to vicinity of dorsal-fin origin vs. not reaching to dorsal-fin origin; maxillary barbels just reaching or slightly surpassing pectoral-fin origin vs. surpassing pectoral-fin origin or even reaching posterior end of gill membrane; principal caudal-fin rays usually 17 vs. 14–15; abdominal vertebrae 23–25 vs. 25–27 (data from Ng & Vidthayanon, 2014).

*Exostoma gaoligongense* can be further distinguished from *E*. *berdmorei* by a shorter snout, snout length 39.7%–53.3% HL vs. 55.5–56.9. It can be further distinguished from *E*. *peregrinator* by a short dorsal-fin base, 8.3%–12.1% SL vs. 12.3–13.8, a shorter snout, snout length 39.7%–53.3% HL vs. 56.1–61.2. It can be further distinguished from *E*. *sawmteai* by pelvic fin reaching anus vs. not reaching, a short dorsal-fin base, 8.3%–12.1% SL vs. 12.4–13.5, and a longer anal fin, 13.1%–15.8% SL vs. 7.5–10.4.

*Exostoma gaoligongense* can be further distinguished from *E*. *barakense*, *E*. *effrenum* and *E*. *tenuicaudata* by gill opening extending to ventral surface of the body vs. not reaching, and a shorter snout, snout length 39.7%–53.3% HL vs. 57.3–64.6. It can be further distinguished from *E*. *barakense* by pelvic fin reaching anus vs. not reaching. It can be further distinguished from *E*. *effrenum* by emarginate caudal fin vs. forked, shorter nasal barbels, reaching or surpassing anterior edge of eye vs. reaching nearly to posterior orbital margin, shorter dorsal-fin base, 8.3%–12.1% SL vs. 12.9–15.3, longer adipose-fin base, 31.7%–45.2% SL vs. 25.8–29.0. It can be further distinguished from *E*. *tenuicaudata* by pelvic fin reaching anus vs. not reaching, a longer anal fin, 13.1%–15.8% SL vs. 10.0–13.0, stouter caudal peduncle, its length 18.8%–22.3% SL vs. 26.3–28.0, its depth 7.3%–10.8% SL vs. 3.6–4.7, deeper body, body depth at anus 11.3%–16.9% SL vs. 9.6–11.3, wider interorbital width, 27.5%–35.1% HL vs. 24.4–26.4.

*Exostoma gaoligongense* shares gill opening extending to ventral surface of the body with *E*. *labiatum* and *E*. *stuarti*. It can be further distinguished from *E*. *labiatum* and *E*. *stuarti* by adipose fin confluent with the upper procurrent caudal-fin rays vs. free from it. It can be further distinguished from *E*. *labiatum* by branched pectoral-fin rays 9–11 vs. 12–13. *Exostoma stuarti* has a distinctly arched dorsum and only three branched anal-fin ray relative to all congeners. *Exostoma gaoligongense* can be further distinguished from *E*. *stuarti* in pelvic fin reaching anus vs. not reaching, shorter caudal-peduncle, it length 18.8%–22.3% SL vs. 14.2–15.0, and relative more anterior position of anal-fin, anal to caudal distance/pelvic to anal distance 88.2%–116.7% vs. 79.6–82.6.

Hora & Silas (1952) recognized five valid species among the nominal species of *Exostoma* at that time, and of the five, one is included in a different genus now, namely *Pseudexostoma yunnanensis*. *Exostoma labiatum* was described from the Mishmis Hills of Assam [larger area of earlier times], India (McClelland, 1842) or known as Danbajiang, Tibet, China (Wu & Wu, 1992), and later was reported from other sites in the Brahmaputra and Chindwin river drainages of India (Hora & Mukerji, 1935; Hora & Silas, 1952), Brahmaputra River drainage of Tibet (Wu et al., 1981; Wu, 1985; Wu & Wu, 1992), and rivers in the Irrawaddy River drainage of Yunnan (Chu & Mo, 1999; Chu et al., 1990; Wu et al., 1981). *Exostoma berdmorei* was described from Tenasserim, southern Myanmar (Blyth, 1860; Hora & Silas, 1952), the second collection was from Dawna Hills, Kawkareik, Sukli, Kayin State, Myanmar, in 31 October 1934 (Fishbase-on-line). A small collection of Dr. Tyson Roberts kept in CAS from Salween drainage was examined for this study. From its forked caudal-fin, apparent small gill opening and only 14 branched caudal-fin rays, these fishes match very well characters of *E*. *berdmorei* in Hora & Silas (1952). It differs from description of Hora & Silas (1952) in the following characters: nasal barbel reaches eye or not vs. not reach; body depth 5.9–6.7 in SL vs. 9–10; HL 4.1–4.4 in SL vs. 5.0–5.17; snout length 1.7–1.9 post-orbital length vs. just larger than. These fishes match very well the redescription of *E*. *berdmorei* in Ng & Vidthayanon (2014), so we treat these specimens as *E*. *berdmorei*.

*Exostoma stuarti* was described from the Nam-Yak River at Tanja in northern Myanmar (from a single specimen) and later was reported from the Chatrickong River drainage in Manipur, India (Vishwanath et al., 1998; Keishing & Vishwanath, 1999). The first author collected 9 specimens from Tanjar Stream, Putao, 26 December 2015. The data of *E*. *stuarti* in this study was based on this collection. The description of *E*. *vinciguerrae* was based on one specimen from the Putao Plains, Kachin, northern Myanmar (Regan, 1905) and later was reported from Pazi Monghong, Hsipi State, and northern Shan States, northeastern Myanmar and Manipur, India (Hora and Silas, 1952; Vishwanath et al., 1998). Five collections from Zeyar and Ponyin streams, from 9 to 14 December, 2015, Hponkanrazi Wildlife Sanctuary, Malihka Drainage, Putao, collected by the first author and others were identified as *E*. *vinciguerrae*.

It seems Hora (Hora & Mukerji, 1935) assigned both *Exostoma* from Brahamaputra and Chindwin drainages (a big upper tributary of Irrawaddy) to *E*. *labiatum*, but later he (Hora, 1923; Hora & Silas, 1952) defined those from Brahamaputra as *E*. *labiatum*, while those from Upper Burma (Irrawaddy) as *E.* *vinciguerrae*.

Wu et al. (1981) pointed out that according to the key of Hora & Silas (1952), *E*. *labiatum* and *E*. *vinciguerrae* differed in the shape of caudal fin (deeply emarginate vs. shallow emarginate) and number of branched pectoral-fin rays (12 vs. 10). Wu et al. (1981) compared specimens from Chayu (=Zayu) and Motuo (=Medog), the upper Brahmaputra River drainage in Tibet with some from Tengchong and Yingjiang, the Irrawaddy River drainage in Yunnan and found there were no prominent differences between them. Within-population variability in the caudal fin concavity (varied from shallow to deep) and branched pectoral-fin rays (mostly 10, a few 11) indicated that these characters will not distinguish the species reliably. From their observations, Wu et al. (1981) concluded that *E*. *vinciguerrae* was most likely a synonym of *E*. *labiatum*. Chu et al. (1990) pointed out that in specimens from the Irrawaddy River drainage in Yunnan the adipose fin can be confluent with the caudal fin or free from it; they questioned the use of caudal fin concavity and confluence of adipose and caudal fins by Hora & Silas (1952) and treated both *E*. *vinciguerrae* and *E*. *stuarti* as synonyms of *E*. *labiatum*.

Recent workers (Lalramliana et al., 2015; Ng & Vidthayanon, 2014; Talwar & Jhingran, 1991; Tamang et al., 2015; Vishwanath & Joyshree, 2007) all treated *E*. *labiatum* and *E*. *vinciguerrae* as distinct species. Lalramliana et al. (2015) pointed out "material from the Brahmaputra River drainage in Arunachal Pradesh and Nagaland identified as *E*. *labiatum* by Hora & Silas (1952) differs from the holotype of *E*. *labiatum* in having the adipose fin confluent with (vs. separate from) the upper procurrent caudal-fin rays, suggesting that it may represent an unnamed species". Seeing discrepancy in description of *E*. *labiatum* between Wu & Wu (1992) from upper Brahmaputra and Indian ones from lower, it implies that the identification of *E*. *labiatum* from both upper Brahmaputra and Irrawaddy drainages of China should be verified carefully in the future. Herein we follow these recent workers and confine *E*. *vinciguerrae* (and *E*. *chaudhurii*) in Malihka Drainage of Myanmar, and confine *E*. *labiatum* in Brahmaputra Drainage.

By comparison of available data from our material and literatures (Tamang et al., 2015; Vishwanath & Joyshree, 2007), *E*. *labiatum* can be readily distinguished from *E*. *vinciguerrae* by caudal fin deeply emarginate vs. shallow emarginate, branched pectoral-fin rays 12–13 vs. 10–11, adipose-fin base 26.2% SL vs. 28.5–39.3, more slender caudal peduncle, its length 2.6–3.2 times depth vs. 2.1–2.2.

Based on all the results mentioned above, an artificial key to genus *Exostoma* is tentatively provided as follows:

### Key to *Exostoma* species

1 Adipose fin confluent with caudal fin................................. 2

– Adipose fin separated from caudal fin............................... 6

2 Gill opening extending to ventral surface of the body.............. 3

– Gill opening not extending to ventral surface of the body........ 4

3 Pelvic fin not reaching anus; pectoral fin not reaching to dorsal-fin origin; principal caudal-fin rays 14–15............................................*E*. *vinciguerrae* (Irrawaddy Basin)

– Pelvic fin reaching anus; pectoral fin extending to vicinity of dorsal-fin origin origin; principal caudal-fin rays usually 17......................................... *E*. *gaoligongense* (Salween Basin)

4 Caudal fin slightly forked..... *E*. *berdmorei* (Salween Basin)

– Caudal fin lunate................................................................... 5

5 Snout length 48%–55% HL; caudal-peduncle depth 10.1%–11.7% SL..........*E*. *sawmteai* (Surma-Meghna Basin)

– Snout length 56%–61% HL; caudal-peduncle depth 6.2%–8.5% SL..........*E*. *peregrinator* (Chao Phraya Basin)

6 Posterior tip of adipose fin with a distinct incision.......... 7

– Posterior tip of adipose fin without an incision................ 9

7 Caudal peduncle slender, its length 26.3%–28.0% SL, depth 3.6%–4.7% SL.............*E*. *tenuicaudata* (Brahmaputra Basin)

– Caudal peduncle deeper, its length 17.0%–21.3% SL, depth 5.1%–11.1% SL......................................................................8

8 Caudal fin emarginate; body and caudal peduncle deeper, body depth at anus 14.0%–16.5% SL, caudal-peduncle depth 9.5%–11.1% SL.. *E*. *barakense* (Surma-Meghna Basin)

– Caudal fin forked; body and caudal peduncle slender, body depth at anus 10.5%–12.4% SL, caudal-peduncle depth 5.1%–7.0% SL................... *E*. *effrenum* (Chao Phraya Basin)

9 Dorsal profile greatly arched; postdorsal profile severely sloping......................................... *E*. *stuarti* (Irrawaddy Basin)

– Dorsal profile slightly arched; postdorsal profile gently sloping.................................*E*. *labiatum* (Brahmaputra Basin)

## COMPARATIVE MATERIAL EXAMINED

***Exostoma berdmorei***: THAILAND: SALWEEN BASIN: NIFI uncataloged, 5 ex., labeled as *Oreoglanis*, kept in CAS, Salween Basin/Maejala O. (Tributary of Menam Moei/Tak province (Ta Song Yang Dist.), collected by S. Ukkotawerat, no date.

***Exostoma labiatum***: CHINA: BRAHMAPUTRA BASIN: IHB 13800117 (73 Ⅶ 0074–76), 3 ex., 50.29–67.35 mm SL, Angqu River, Ciba, Chayu, Tibet, July 1973; IHB 13800143 (74 Ⅶ 2096, 74 Ⅸ 2171, 2174, 2179), 4 ex., 55.77–61.44 mm SL, Beiben, Motuo, Tibet, May 1974; IHB 13800142 (74 Ⅸ 2175, 2186–2187), 3 ex., 54.70–56.05 mm SL, Beiben, Motuo, Tibet, August 1974.

***Exostoma stuarti***: MYANMAR: MALIHKA BASIN: KIZ–CXY20150304–305, uncataloged, 3 ex., 33.44–38.16 mm SL, Tanjar Stream, a tributary of Mali Hka Drainage, Hponkanrazi Wildlife Sanctuary, Kachin State, Mynamar, collected by X. Y. Chen, T. Qin, S. S. Shu and Y. M. Kaw, 26 December 2015.

***Exostoma vinciguerrae***: MYANMAR: MALIHKA BASIN: SEABRI 20150255–258, 22 ex., Zeyar stream near Zeyar Dan Village, Hponkanrazi Wildlife Sanctuary (N27°34x12.08˝, E97°06x02.73˝), collected by X. Y. Chen, T. Qin, S. S. Shu and Y. M. Kaw, 9 December 2015; SEABRI 20150371–374, 4 ex., right tributary of upper tributary of Ponyin Stream around Camp 1 of Hponkanrazim (N27°35x43.87˝, E96°59x46.33˝), collected by T. Qin and S. S. Shu, 11 December 2015; SEABRI 20150383–393, 11 ex., upper tributary of Ponyin Stream around Camp 1 of Hponkanrazi, collected by T. Qin and S. S. Shu, 12 December 2015; SEABRI 20150396–399, 4 ex., left tributary of upper tributary of Ponyin Stream around Camp 1 of Hponkanrazi, collected by T. Qin and S. S. Shu, 13 December 2015; SEABRI S20150422 (CXY20150145), 1 ex., Ponyin Stream near Zeyar Dan Village, Hponkanrazi Wildlife Sanctuary (N27°33x51.77˝, E97°05x25.11˝), collected by X. Y. Chen, T. Qin, S. S. Shu and Y. M. Kaw, 14 December 2015.

## ACKNOWLEDGEMENTS

We thank Zheng-Bo Li, Hua Yang, Wei Zhao, Sheng-Wei Yan and Hong-Sen Fan, Gaoligong Mountain National Nature Reserve Management Bureau; Zhi-Sheng Wang, Nangun River National Nature Reserve Management Bureau; Zi-Ming Chen, De-Ping Kong, Xiao-Fu Pan, Jing-Hui Chen, Jian Yang, Fei Wu, Shu-Sen Shu, Tao Qin and Lu-lu Xu, KIZ, and Daw Yunn Mi Mi Kyaw, Forest Research Institute, Myanmar, for their help in field work and other aspects. XYC thanks Li-Na Du, Gui-Hua Cui, Rui Ming, and Meng-Ni He, KIZ and De-Kui He, IHB, for convenience in checking specimens. We especially thank Jun-Xing Yang, KIZ, for help in many aspects, J. Wilkinson, CAS, for providing excellent logistical support throughout the Fall 2003 expedition, and J. Fong, CAS, for preparing radiograph. We thank two anonymous reviewers for inspiring and helpful comments.
